# Calculating the power to examine treatment‐covariate interactions when planning an individual participant data meta‐analysis of randomized trials with a binary outcome

**DOI:** 10.1002/sim.9538

**Published:** 2022-08-05

**Authors:** Richard D. Riley, Miriam Hattle, Gary S. Collins, Rebecca Whittle, Joie Ensor

**Affiliations:** ^1^ Centre for Prognosis Research, School of Medicine Keele University Keele Staffordshire UK; ^2^ Centre for Statistics in Medicine, Nuffield Department of Orthopaedics, Rheumatology and Musculoskeletal Sciences University of Oxford Oxford UK; ^3^ NIHR Oxford Biomedical Research Centre Oxford University Hospitals NHS Foundation Trust Oxford UK

**Keywords:** individual participant data (IPD), meta‐analysis, power, treatment effect modifier, treatment‐covariate interaction

## Abstract

Before embarking on an individual participant data meta‐analysis (IPDMA) project, researchers and funders need assurance it is worth their time and cost. This should include consideration of how many studies are promising their IPD and, given the characteristics of these studies, the power of an IPDMA including them. Here, we show how to estimate the power of a planned IPDMA of randomized trials to examine treatment‐covariate interactions at the participant level (ie, treatment effect modifiers). We focus on a binary outcome with binary or continuous covariates, and propose a three‐step approach, which assumes the true interaction size is common to all trials. In step one, the user must specify a minimally important interaction size and, for each trial separately (eg, as obtained from trial publications), the following aggregate data: the number of participants and events in control and treatment groups, the mean and SD for each continuous covariate, and the proportion of participants in each category for each binary covariate. This allows the variance of the interaction estimate to be calculated for each trial, using an analytic solution for Fisher's information matrix from a logistic regression model. Step 2 calculates the variance of the summary interaction estimate from the planned IPDMA (equal to the inverse of the sum of the inverse trial variances from step 1), and step 3 calculates the corresponding power based on a two‐sided Wald test. Stata and R code are provided, and two examples given for illustration. Extension to allow for between‐study heterogeneity is also considered.

## 1 INTRODUCTION

Individual participant data (IPD) meta‐analysis projects herald much promise,^1^ but are potentially time‐consuming, often taking upwards of 2 years to engage with trial investigators; to obtain, clean, harmonize and meta‐analyze the IPD; and to publish and disseminate results. Therefore, before embarking on an IPD meta‐analysis project, researchers and funders may want to be reassured that the time and resources required are worth their investment. This should include consideration of how many trials are likely to provide their IPD and, based on this, estimation of the potential power of the planned IPD meta‐analysis.^2^

In our experience, power calculations and sample size justifications are rarely considered in protocols or publications of IPD meta‐analysis projects. It might be argued that obtaining IPD is almost always worth the investment, in order to best appraise, synthesize, and summarize the existing evidence. However, if researchers knew before IPD collection that their planned IPD meta‐analysis may have, say, only 30% power to detect a clinically important effect, then they might reconsider whether the project should be initiated. Conversely, if a planned IPD meta‐analysis has a potential power of over 80%, then researchers and funders would be more reassured that the required resources are worth investment.

In this article, we propose how to estimate the power of a planned IPD meta‐analysis project, in advance of IPD collection, where the primary objective is to synthesize IPD from randomized trials to examine treatment‐covariate interactions at the participant level (also known as effect modifiers or moderators). The drive to identify treatment‐covariate interactions stems from the idea of stratified (personalized or precision) medicine, where treatment decisions are tailored to each individual conditional on their covariate values. The availability of IPD from existing trials improves the ability and power to examine treatment‐covariate interactions, compared to single trials or a traditional meta‐analysis of published aggregate data. Hence, the primary objective of many IPD meta‐analysis projects is to examine treatment‐covariate interactions, especially those synthesizing IPD from randomized trials.

Previous work in this area has focused mainly on continuous outcomes,^2^, ^3^, ^4^ or simulation‐based approaches,^5^, ^6^ but here we focus on analytic solutions for binary outcomes. In Section 2 we introduce a two‐stage IPD meta‐analysis framework for the synthesis of interaction estimates from randomized trials. However, before IPD are obtained, we assume estimates of the desired interactions are not available from the trials to be included in the IPD meta‐analysis project (if they were, the need for IPD may be unjustified). Rather, we assume users can extract basic aggregate data from each trial, such as the number of participants and events, the proportion in each category (eg, proportion male) for binary covariates, and the mean and SD of continuous covariates. In Section 3 we show how this aggregate data can be used to approximate the variance of each trial's interaction estimate from a logistic regression model, adapting analytic (closed‐form) solutions proposed by Demidenko.^7^ Section 4 explains how to subsequently use these trial variances to calculate the corresponding power of the planned IPD meta‐analysis project. Section 5 illustrates the proposal with two examples, and our results are compared to an approximate approach proposed by Kovalchick and Cumberland.^8^ Section 6 considers extension to allow for between‐trial heterogeneity in the true interaction size, and how to focus on precision rather than power. Finally, Section 7 concludes with discussion. Stata code is provided in the Supplementary material, and R code is available at www.github.com/gscollins1973.

## 2 A TWO‐STAGE APPROACH TO ESTIMATING A TREATMENT‐COVARIATE INTERACTION IN AN IPD META‐ANALYSIS WITH A BINARY OUTCOME

In this article we focus on a two‐stage IPD meta‐analysis for summarizing a treatment‐covariate interaction. In the first stage, the treatment‐covariate interactions are estimated using the IPD in each trial separately; in the second stage, these interaction estimates are pooled using a chosen meta‐analysis model.^3^ This two‐stage approach can be implemented using *ipdmetan* in Stata.^9^ By only pooling interaction estimates derived from within‐trial information (ie, based at the participant level), this approach automatically avoids trial‐level confounding and aggregation bias that may occur in meta‐regression based on across‐trial information,^10^, ^11^ in a multivariate meta‐analysis that jointly synthesizes interactions and reference treatment effects,^4^ or in one‐stage IPD meta‐analysis models that do not separate out within‐trial and across‐trial relationships.^4^, ^12^ Our website (www.ipdma.co.uk) provides examples of statistical code to implement two‐stage and one‐stage approaches, and broad discussion of modeling interactions in IPD meta‐analysis is given elsewhere.^1^, ^4^

We now provide details on the two‐stage approach for estimating a treatment‐covariate interaction from an IPD meta‐analysis of S trials with a binary outcome. This provides the foundation for our power calculations which follow in subsequent sections.

### 2.1 First‐stage

Consider IPD from a parallel group trial, comparing a treatment ($x_{\mathrm{ij}}=1$) to a control ($x_{\mathrm{ij}}=0$). Let $z_{\mathrm{ij}}$ be a participant‐level covariate (eg, the age of participant j in trial i), observed for all participants in each trial, and consider that a binary outcome is of interest (ie, $y_{\mathrm{ij}}=0$ or 1, where 0 denotes no event and 1 denotes an event occurred), such as whether pre‐eclampsia occurred during pregnancy. To estimate the treatment‐covariate interaction in each trial separately, a logistic regression could be fitted as follows,

(1) $$\begin{array}{cc} & y_{\mathrm{ij}}∼\text{Bernoulli}\left(p_{\mathrm{ij}}\right)\\ & \ln\left(\frac{p_{\mathrm{ij}}}{1-p_{\mathrm{ij}}}\right)=\alpha_{i}+\beta_{i}x_{\mathrm{ij}}+\gamma_{i}z_{\mathrm{ij}}+\lambda_{i}x_{\mathrm{ij}}z_{\mathrm{ij}} \end{array}$$

where $p_{\mathrm{ij}}$ is the probability of the outcome event for participant j in trial i. The trial subscript i is not strictly required, but we include it to emphasise that the logistic regression is applied to each of the i=1toS trials separately, leading to S estimates of each parameter (one for each trial). Equation (1) is usually estimated using maximum likelihood estimation, and this is our focus here, although adaptations are potentially important if sparse data are a concern.^13^, ^14^

The treatment‐covariate interaction term, $\lambda_{i}$, indicates the expected change in treatment effect (log odds ratio) for a one‐unit increase in $z_{\mathrm{ij}}$ for trial i, and is adjusted for the prognostic effect ($\gamma_{i}$) of the covariate of interest ($z_{\mathrm{ij}})$ and the reference treatment effect ($\beta_{i})$. Other prognostic factors could also be adjusted for, but we do not consider this here. For a continuous covariate, Equation (1) assumes the effect of the interaction is linear (although extension to non‐linear trends is important in practice^4^, ^15^).

### 2.2 Second stage

The first stage produces S estimates of the treatment‐covariate interaction (${\hat{\lambda }}_{i}$) and its variance ($\mathrm{var}\left({\hat{\lambda }}_{i}\right)$), one pair for each trial in the IPD meta‐analysis. In the second stage, the ${\hat{\lambda }}_{i}$ values are synthesised using either a common‐effect model (ie, the true interaction is assumed the same in all trials, denoted by λ),

(2) $${\hat{\lambda }}_{i}∼N\left(\lambda ,\mathrm{var}\left({\hat{\lambda }}_{i}\right)\right)$$

or a random‐effects model (ie, the true interactions in the trials are assumed randomly drawn from a normal distribution, with a mean of λ and between‐study variance of $\tau^{2}$):

(3) $$\begin{array}{cc} & {\hat{\lambda }}_{i}∼N\left(\lambda_{i},\mathrm{var}\left({\hat{\lambda }}_{i}\right)\right)\\ & \lambda_{i}∼N\left(\lambda ,\tau^{2}\right) \end{array}$$

Maximum likelihood estimation can be used to fit Equation (2), whereas restricted maximum likelihood (REML) is recommended to fit model Equation (3).^16^ The summary estimate of λ will be a weighted average, and it summarises the difference in the expected treatment effect for two participants who differ in $z_{\mathrm{ij}}$ by one unit. That is, λ represents the summary difference in log odds ratios (ie, exp(λ) gives a ratio of odds ratios) for two individuals that differ in $z_{\mathrm{ij}}$ by one‐unit. The smaller the $\mathrm{var}\left({\hat{\lambda }}_{i}\right)$ for a trial, the more weight it has in the meta‐analysis.

For the common‐effect model, the variance of the summary interaction estimate is

(4) $$\mathrm{var}\left(\hat{\lambda }\right)=\frac{1}{\sum_{i=1}^{S}{\left(\mathrm{var}\left({\hat{\lambda }}_{i}\right)\right)}^{-1}},$$

where S is the total number of trials in the IPD meta‐analysis.

For the random‐effects model, the variance of the summary interaction estimate is,

(5) $$\mathrm{var}\left(\hat{\lambda }\right)=\frac{1}{\sum_{i=1}^{S}{\left(\mathrm{var}\left({\hat{\lambda }}_{i}\right)+{\hat{\tau }}^{2}\right)}^{-1}},$$

where ${\hat{\tau }}^{2}$ is the (REML) estimated between‐trial variance in interaction.

In order to consider the potential power of an IPD meta‐analysis project, we need to ascertain the potential value of $\mathrm{var}\left(\hat{\lambda }\right)$ in advance. Fundamentally this depends on the trial variances ($\mathrm{var}\left({\hat{\lambda }}_{i}\right))$, and so the next section proposes how these may be ascertained in advance of IPD collection.

## 3 CALCULATING THE VARIANCE OF A TREATMENT‐COVARIATE INTERACTION ESTIMATE FOR A BINARY OUTCOME IN A SINGLE TRIAL

In this section we describe analytical (closed‐form) solutions for $\mathrm{var}\left({\hat{\lambda }}_{i}\right)$ in a single randomized trial, based on Fisher's Information matrix. Such solutions are challenging to obtain as, unlike for continuous outcomes, $\mathrm{var}\left({\hat{\lambda }}_{i}\right)$ will be correlated with the value of ${\hat{\lambda }}_{i}$ itself. The reason is that for generalized linear models such as the logistic regression model in Equation (1), each participant‐level variance is a function of the participant's predicted outcome values from the fitted model. In other words, rather than considering one variance term per trial for a continuous outcome (ie, a single residual variance, $\sigma_{i}^{2}$, or one residual variance term per treatment group), for binary outcomes a separate variance term ($\sigma_{\mathrm{ij}}^{2}$) is required for each participant, conditional on their covariate values. Specifically, for binary outcomes, a participant's response variance is $p_{\mathrm{ij}}\left(1-p_{\mathrm{ij}}\right)$ and thus depends on their expected outcome probability ($p_{\mathrm{ij}})$, which is conditional on the baseline risk in the trial and the prognostic effect of any covariates, including the interaction and treatment terms. This makes closed‐form solutions problematic, and so Kovalchik and Cumberland suggested approximating these variances to enable a closed‐form solution (based on matrix algebra, and implemented within the package *ipdmeta* in R).^8^ Their approach requires strong assumptions, such as replacing each participant's $p_{\mathrm{ij}}\left(1-p_{\mathrm{ij}}\right)$ with $p_{i}\left(1-p_{i}\right)$, where $p_{i}$ is the overall outcome risk in the participant's corresponding group within trial i. Such approximations may not be reliable; for example, the error in the approximate closed‐form power estimates of Kovalchik and Cumberland is often over 10%.^8^ Their approach also assumes a one‐stage model that amalgamates within‐trial and across‐trial information,^8^ and so will generally over‐estimate the actual power.

To address these concerns, we propose to rather use and extend the analytic solutions for $\mathrm{var}\left({\hat{\lambda }}_{i}\right)$ derived by Demidenko et al,^7^ which are asymptotically exact based on Fisher's information matrix. We derive this for a binary covariate (Section 3.1) and then for a continuous covariate (Section 3.2). Section 4 utilizes the solutions for the power calculation for IPD meta‐analysis projects.

### 3.1 Binary covariate

Let $z_{\mathrm{ij}}$ be a binary covariate, such as $z_{\mathrm{ij}}$ = 1 for males and $z_{\mathrm{ij}}$ = 0 for females. Then, Demidenko shows that the asymptotic variance of ${\hat{\lambda }}_{i}$ after fitting the logistic regression model in Equation (1) is,^7^

(6) $$\mathrm{var}\left({\hat{\lambda }}_{i}\right)=I_{i}^{-1}\left(4,4\right)/n_{i},$$

where $n_{i}$ is the total sample size of trial i, and $I_{i}^{-1}\left(4,4\right)$ denotes the 4,4 element of the inverse matrix of Fisher's unit information matrix (I) (we use the word “unit” as it is independent of sample size).

Given the design matrix $X={\left(1,x_{\mathrm{ij}},z_{\mathrm{ij}},x_{\mathrm{ij}}z_{\mathrm{ij}}\right)}^{\prime }$, the 4 by 4 unit information matrix for the *i*th trial can be expressed as,

(7) $$\begin{array}{cc} & I_{i}=E_{\left(x,z\right)}\left(\frac{\exp\left(\alpha_{i}+\beta_{i}x_{\mathrm{ij}}+\gamma_{i}z_{\mathrm{ij}}+\lambda_{i}x_{\mathrm{ij}}z_{\mathrm{ij}}\right)}{{\left(1+\exp\left(\alpha_{i}+\beta_{i}x_{\mathrm{ij}}+\gamma_{i}z_{\mathrm{ij}}+\lambda_{i}x_{\mathrm{ij}}z_{\mathrm{ij}}\right)\right)}^{2}}XX^{\prime }\right)\\ & =E_{\left(x,z\right)}\left(\frac{\exp\left(\alpha_{i}+\beta_{i}x_{\mathrm{ij}}+\gamma_{i}z_{\mathrm{ij}}+\lambda_{i}x_{\mathrm{ij}}z_{\mathrm{ij}}\right)}{{\left(1+\exp\left(\alpha_{i}+\beta_{i}x_{\mathrm{ij}}+\gamma_{i}z_{\mathrm{ij}}+\lambda_{i}x_{\mathrm{ij}}z_{\mathrm{ij}}\right)\right)}^{2}}\left[\begin{array}{cccc} 1 & x_{\mathrm{ij}} & z_{\mathrm{ij}} & x_{\mathrm{ij}}z_{\mathrm{ij}}\\ x_{\mathrm{ij}} & x_{\mathrm{ij}} & x_{\mathrm{ij}}z_{\mathrm{ij}} & x_{\mathrm{ij}}z_{\mathrm{ij}}\\ z_{\mathrm{ij}} & x_{\mathrm{ij}}z_{\mathrm{ij}} & z_{\mathrm{ij}} & x_{\mathrm{ij}}z_{\mathrm{ij}}\\ x_{\mathrm{ij}}z_{\mathrm{ij}} & x_{\mathrm{ij}}z_{\mathrm{ij}} & x_{\mathrm{ij}}z_{\mathrm{ij}} & x_{\mathrm{ij}}z_{\mathrm{ij}} \end{array}\right]\right), \end{array}$$

where $E_{\left(x,z\right)}\left(B\right)$ denotes the expected value of B over the joint distribution of x and z. Note that the expected value of a matrix B is formed by the matrix of expected values of the elements of B.

Demidenko shows the unit information matrix can be expanded into a closed‐form solution of^7^:

(8) $$\begin{array}{cc} & I_{i}=\frac{\exp\left(\alpha_{i}\right)}{{\left(1+\exp\left(\alpha_{i}\right)\right)}^{2}}M_{1}\;\mathrm{Pr}\left(x_{\mathrm{ij}}=0,z_{\mathrm{ij}}=0\right)\\ & \begin{array}{c} \;+\;\frac{\exp\left(\alpha_{i}+\beta_{i}\right)}{{\left(1+\exp\left(\alpha_{i}+\beta_{i}\right)\right)}^{2}}M_{2}\;\mathrm{Pr}\left(x_{\mathrm{ij}}=1,z_{\mathrm{ij}}=0\right)\\ \begin{array}{c} \;+\;\frac{\exp\left(\alpha_{i}+\gamma_{i}\right)}{{\left(1+\exp\left(\alpha_{i}+\gamma_{i}\right)\right)}^{2}}M_{3}\;\mathrm{Pr}\left(x_{\mathrm{ij}}=0,z_{\mathrm{ij}}=1\right)\\ \;+\;\frac{\exp\left(\alpha_{i}+\beta_{i}+\gamma_{i}+\lambda_{i}\right)}{{\left(1+\exp\left(\alpha_{i}+\beta_{i}+\gamma_{i}+\lambda_{i}\right)\right)}^{2}}M_{4}\;\mathrm{Pr}\left(x_{\mathrm{ij}}=1,z_{\mathrm{ij}}=1\right), \end{array} \end{array} \end{array}$$

where

$$M_{1}=\left[\begin{array}{cccc} 1 & 0 & 0 & 0\\ 0 & 0 & 0 & 0\\ 0 & 0 & 0 & 0\\ 0 & 0 & 0 & 0 \end{array}\right]\;M_{2}=\left[\begin{array}{cccc} 1 & 1 & 0 & 0\\ 1 & 1 & 0 & 0\\ 0 & 0 & 0 & 0\\ 0 & 0 & 0 & 0 \end{array}\right]\;M_{3}=\left[\begin{array}{cccc} 1 & 0 & 1 & 0\\ 0 & 0 & 0 & 0\\ 1 & 0 & 1 & 0\\ 0 & 0 & 0 & 0 \end{array}\right]\;M_{4}=\left[\begin{array}{cccc} 1 & 1 & 1 & 1\\ 1 & 1 & 1 & 1\\ 1 & 1 & 1 & 1\\ 1 & 1 & 1 & 1 \end{array}\right].$$

Therefore, to derive the unit information matrix after fitting the logistic regression of Equation (1) to a particular trial, we need to specify the estimated values of parameters α,β,γ,andλ alongside the four joint probabilities $\mathrm{Pr}\left(x_{\mathrm{ij}}=a,z_{\mathrm{ij}}=b\right)$, estimated as the proportion of participants in the trial classified as $x_{\mathrm{ij}}=0,z_{\mathrm{ij}}=0\;$; proportion classified as $x_{\mathrm{ij}}=1,z_{\mathrm{ij}}=0$; proportion classified as $x_{\mathrm{ij}}=0,z_{\mathrm{ij}}=1$; and proportion classified as $x_{\mathrm{ij}}=1,z_{\mathrm{ij}}=1$. We can then derive the asymptotic variance of the interaction estimate, using Equation (6) (ie, $\mathrm{var}\left(\hat{\lambda }\right)=I_{i}^{-1}\left(4,4\right)/n_{i}$).

We will adapt this approach (specifically Equation (8) followed by Equation (6)) when extending to the IPD meta‐analysis setting in Section 4, to estimate the potential $\mathrm{var}\left({\hat{\lambda }}_{i}\right)$ of a binary covariate for each trial planned to be in the IPD meta‐analysis project.

**FIGURE 1:**
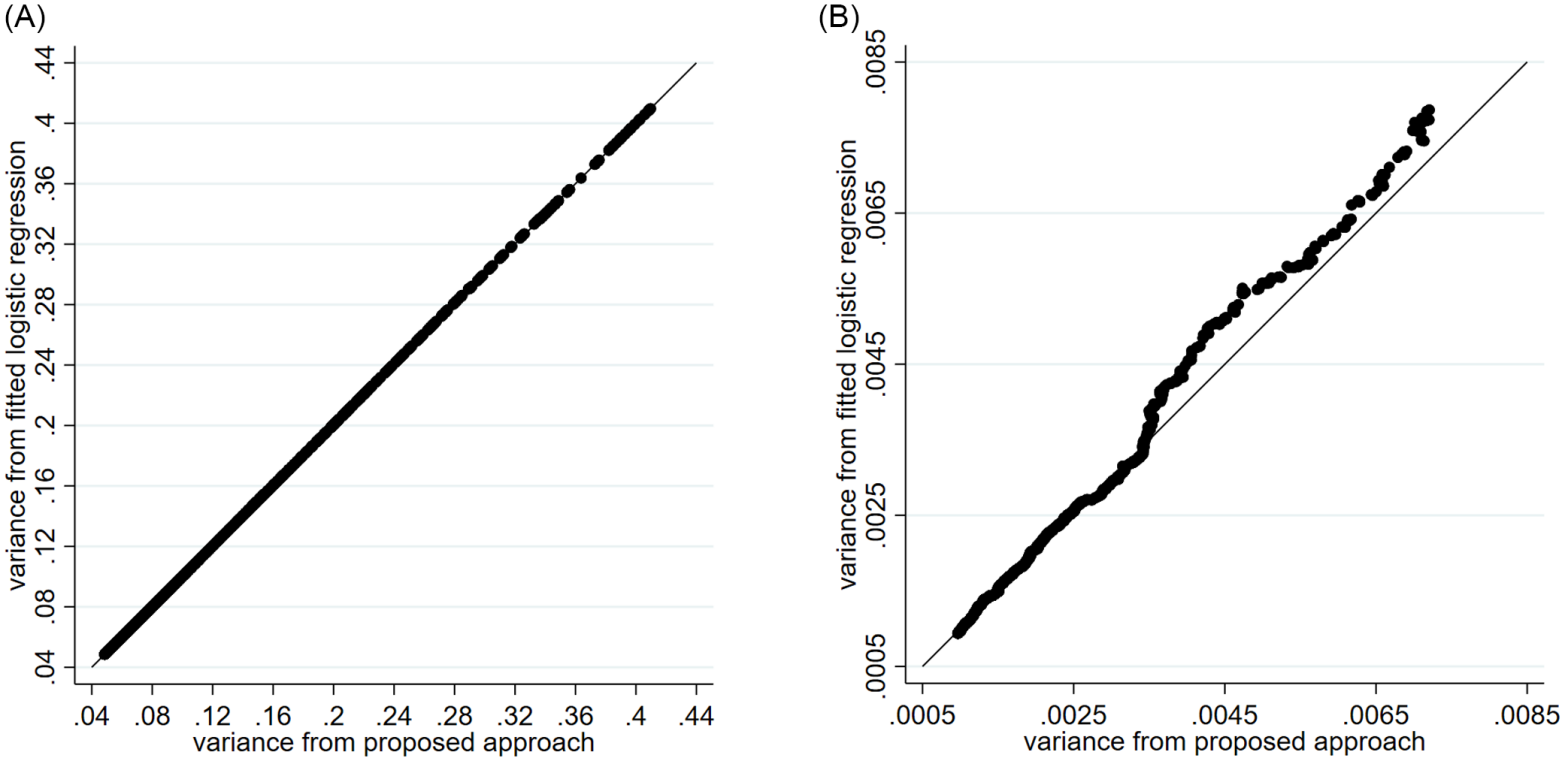
Comparison of the variance of a treatment‐covariate interaction from the randomized trial of Wilson et al,^17^ as estimated from (i) fitting a logistic regression model to the IPD (via maximum likelihood estimation using the logit command in Stata) or (ii) our approach (ie, for a binary covariate Equation (8) followed by Equation (6); for a continuous covariate, Equation (9) followed by Equation (6)) applied to the aggregate data assuming the same estimates of $\alpha_{i}$, $\beta_{i}$, $\gamma_{i}$, and $\lambda_{i}$ as the logistic regression estimates. Stata code is available in the Supplementary material. Each point corresponds to a different sample size, ranging from 1739 participants (far left) to 200 participants (far right). (A) Binary covariate—sex. (B) Continuous covariate—age, assuming it was normally distributed based on the observed mean and SD for each trial

To demonstrate the robustness of this approach, we used IPD from a randomized trial that assessed the effects of an internet‐accessed sexually transmitted infection (e‐STI) testing and results service (SH:24) on uptake of STI testing, compared to usual care. The IPD for the trial are freely available in the supplementary material of Wilson et al,^17^ for which we kindly thank the authors, and a primary outcome of interest was self‐reported STI testing at 6 weeks. We examined the variance of the treatment‐age interaction estimated from (i) fitting a logistic regression model (via maximum likelihood estimation) or (ii) our approach (ie, Equation (8) followed by Equation (6)) assuming the same estimates of $\alpha_{i}$, $\beta_{i}$, $\gamma_{i},$ and $\lambda_{i}$ as the logistic regression estimates. We did this for the full dataset (1739 participants: 921 intervention group, 818 usual care group) and then for sequentially lower sample sizes (selected at random) down to 200 participants. The Stata simulation code is provided in the Supplementary material, and the findings are shown in Figure 1A, which confirm that the proposed approach gives variances of interaction estimates identical to those obtained from fitting a logistic regression model when examining a binary covariate.

### 3.2 Continuous covariate

We now extend the work of Demidenko to a continuous covariate ($z_{\mathrm{ij}})$,^7^ again utilizing the solution of Equation (6) that $\mathrm{var}\left({\hat{\lambda }}_{i}\right)=I_{i}^{-1}\left(4,4\right)/n_{i}$. For a continuous covariate, the Fisher's unit information matrix for the logistic regression model in Equation (1) can be written as:

(9) $$\begin{array}{cc} & I_{i}=E_{\left(x,z\right)}\left(\frac{\exp\left(\alpha_{i}+\beta_{i}x_{\mathrm{ij}}+\gamma_{i}z_{\mathrm{ij}}+\lambda_{i}x_{\mathrm{ij}}z_{\mathrm{ij}}\right)}{{\left(1+\exp\left(\alpha_{i}+\beta_{i}x_{\mathrm{ij}}+\gamma_{i}z_{\mathrm{ij}}+\lambda_{i}x_{\mathrm{ij}}z_{\mathrm{ij}}\right)\right)}^{2}}XX^{\prime }\right)\\ & \begin{array}{c} \;=E_{\left(x,z\right)}\left(\frac{\exp\left(\alpha_{i}+\beta_{i}x_{\mathrm{ij}}+\gamma_{i}z_{\mathrm{ij}}+\lambda_{i}x_{\mathrm{ij}}z_{\mathrm{ij}}\right)}{{\left(1+\exp\left(\alpha_{i}+\beta_{i}x_{\mathrm{ij}}+\gamma_{i}z_{\mathrm{ij}}+\lambda_{i}x_{\mathrm{ij}}z_{\mathrm{ij}}\right)\right)}^{2}}\left[\begin{array}{cccc} 1 & x_{\mathrm{ij}} & z_{\mathrm{ij}} & x_{\mathrm{ij}}z_{\mathrm{ij}}\\ x_{\mathrm{ij}} & x_{\mathrm{ij}}^{2} & x_{\mathrm{ij}}z_{\mathrm{ij}} & x_{\mathrm{ij}}^{2}z_{\mathrm{ij}}\\ z_{\mathrm{ij}} & x_{\mathrm{ij}}z_{\mathrm{ij}} & z_{\mathrm{ij}}^{2} & x_{\mathrm{ij}}z_{\mathrm{ij}}^{2}\\ x_{\mathrm{ij}}z_{\mathrm{ij}} & x_{\mathrm{ij}}^{2}z_{\mathrm{ij}} & x_{\mathrm{ij}}z_{\mathrm{ij}}^{2} & x_{\mathrm{ij}}^{2}z_{\mathrm{ij}}^{2} \end{array}\right]\right)\\ \;=E_{\left(x,z\right)}\left(B\right), \end{array} \end{array}$$

where B is a 4 by 4 matrix.

The expected value ($E_{\left(x,z\right)}\left(B\right))$ depends on the joint distribution of the continuous covariate and the treatment/control group allocation (ie, joint distribution of x and z), and also the values of the logistic regression parameters ($\alpha_{i},\beta_{i},\gamma_{i},\lambda_{i})$ themselves. For this reason, and unlike for a binary covariate, it is not possible to modify Equation (9) into a closed‐form solution for $I_{i}$. However, one way to derive $E_{\left(x,z\right)}\left(B\right)$ post estimation, is to calculate each of the 16 components of B for each participant in the trial using the estimated logistic regression parameters, and then their means provide their expected values and thus form $I_{i}$. We can then derive the asymptotic variance of the interaction estimate, using Equation (6) (ie, $\mathrm{var}\left({\hat{\lambda }}_{i}\right)=I_{i}^{-1}\left(4,4\right)/n_{i}$).

We will adapt this approach (specifically Equation (9) followed by Equation (6)) when extending to the IPD meta‐analysis setting in the next section, to estimate the potential $\mathrm{var}\left({\hat{\lambda }}_{i}\right)$ for each trial planned to be in the IPD meta‐analysis project, based on aggregate data available for each trial in terms of the number of participants, events and an assumed covariate distribution. To demonstrate the robustness of this approach, we again used the randomized trial of Wilson et al,^17^ now considering the estimated variance of a treatment‐age interaction from (i) fitting a logistic regression model with (ii) our approach (ie, Equation (9) followed by Equation (6)) assuming the same estimates of $\alpha_{i}$, $\beta_{i}$, $\gamma_{i},$ and $\lambda_{i}$ as the logistic regression estimates, but with the age covariate simulated based on an assumed normal distribution (with means and SDs matching those observed in the IPD for each group). The results are shown in Figure 1B, for trial sample sizes from 200 to 1739 as before. Unlike in the binary covariate situation, our proposal is no longer exact, due to the assumed distribution of the covariate being an approximation. However, it still works well in this example, as the variance estimates for the treatment‐age interaction from our proposal are very similar to those from fitting the logistic regression model to the IPD directly. This is despite the observed distribution of age not being particularly close to a normal distribution (Supplementary Material).

## 4 CALCULATING THE POWER OF A POTENTIAL IPD META‐ANALYSIS PROJECT TO ESTIMATE A TREATMENT‐COVARIATE INTERACTION WITH A BINARY OUTCOME

We return to the main focus of our article: how to calculate the power of an IPD meta‐analysis project aiming to estimate a treatment‐covariate interaction for a binary outcome. We reiterate that our aim is to do this *in advance* of IPD collection and we assume that trials from which IPD are desired have not reported interaction estimates and their variances previously. The overall power of the IPD meta‐analysis is not simply the sum of the power of each individual trial, but rather is a function of the $\mathrm{var}\left({\hat{\lambda }}_{i}\right)$ for the trials (the estimated variances of the trial‐specific interaction estimates), which defines the variance of the summary interaction estimate from the meta‐analysis.

We outline a three‐step process. Step 1 describes how to derive an estimate of $\mathrm{var}\left({\hat{\lambda }}_{i}\right)$ for each trial using routinely reported aggregate data from trial publications, alongside assumptions about effect sizes (in particular, the assumed magnitude of the treatment‐covariate interaction). Step 2 takes these variances and uses them to derive an estimate of the meta‐analysis summary result for the treatment‐covariate interaction. Then, third, the power of the planned IPD meta‐analysis is derived based on these previous values. Stata code to implement the approach is provided in the Supplementary material, and takes just a few seconds to run for a binary covariate, and less than a few minutes for a continuous covariate in the examples provided. R code is available at www.github.com/gscollins1973.

### 4.1 Step 1: Estimate the variance of the treatment‐covariate interaction separately for each trial in the planned IPD meta‐analysis

#### 4.1.1 Binary covariate

For each trial promising their IPD (or for which IPD are sought), the first step is to apply Equation (8) followed by Equation (6) to obtain an estimate of $\mathrm{var}\left({\hat{\lambda }}_{i}\right)$.

To implement the approach, we need to obtain the aggregate data from each trial listed in Table 1. Some of the items listed can be derived from other aggregate data listed, but all items are included for completeness. This set of aggregate data are usually available from trial publications, especially for commonly reported baseline covariates like sex (eg, in a table summarizing baseline characteristics per group, often referred to as “Table 1”), but if not, the original trial investigators could be contacted to provide this information. If only the overall proportion with $z_{\mathrm{ij}}$ = 1 (or = 0) is available, then, as these are randomized trials, it could be assumed that the same proportion occurs in both treatment and control groups.

**TABLE 1:** Aggregate data required from each trial to implement the proposed power calculation for the interaction between treatment effect and a binary covariate

Total participants in the trial ($n_{i})$
Total participants in control group ($n_{Ci})$
Total participants in treatment group ($n_{Ti})$
Number of outcome events in the control group ($e_{Ci})$
Number of outcome events in the treatment group ($e_{Ti})$
Proportion of patients in the trial with $x_{\mathrm{ij}}$ = 0 and $z_{\mathrm{ij}}$ = 0 $\left(\mathrm{Pr}\left(x_{\mathrm{ij}}=0,z_{\mathrm{ij}}=0\right)\right)$
Proportion of patients in the trial with $x_{\mathrm{ij}}$ = 0 and $z_{\mathrm{ij}}$ = 1 $\left(\mathrm{Pr}\left(x_{\mathrm{ij}}=0,z_{\mathrm{ij}}=1\right)\right)$
Proportion of patients in the trial with $x_{\mathrm{ij}}$ = 1 and $z_{\mathrm{ij}}$ = 0 $\left(\mathrm{Pr}\left(x_{\mathrm{ij}}=1,z_{\mathrm{ij}}=0\right)\right)$
Proportion of patients in the trial with $z_{\mathrm{ij}}$ = 1 and $x_{\mathrm{ij}}$ = 1 $\left(\mathrm{Pr}\left(x_{\mathrm{ij}}=1,z_{\mathrm{ij}}=1\right)\right)$

We also need to make assumptions about the values of the logistic regression parameters in each of the trials ($\alpha_{i},\beta_{i},\gamma_{i},\lambda_{i})$. Without loss of generalisability, let us assume $z_{\mathrm{ij}}$ will be centered by its trial‐specific mean when fitting the logistic regression of Equation (1). This does not change the interpretation of the interaction term ($\lambda_{i})$ or the prognostic effect of the covariate ($\gamma_{i})$, but it helps to interpret the two other parameters. That is, $\alpha_{i}$ now becomes the log‐odds of the outcome in trial i in the control group for a participant with the mean value of $z_{\mathrm{ij}}$. This can be approximated by the overall log‐odds of the outcome event in the control group of trial i, which can be derived from $\log(e_{Ci}/$($n_{Ci}-e_{Ci}))$. The treatment effect ($\beta_{i}$) for a participant with the mean value of $z_{\mathrm{ij}}$ can be approximated by the overall treatment effect (log odds ratio) in the trial. If not reported directly, this log odds ratio can be calculated manually from $n_{Ci}$, $n_{Ti}$, $e_{Ci},$ and $e_{Ti}$.

In terms of the prognostic effect ($\gamma_{i})$ of the covariate, we suggest assuming this is zero for simplicity, or considering a range of possible values (see examples later). For the key parameter ($\lambda_{i})$, we suggest identifying a minimally important value via discussion with clinical experts within the IPD meta‐analysis project team. It is simplest to assume $\lambda_{i}$ is common for all trials (ie, $\lambda_{i}=\lambda$), although we discuss extensions to allow for between‐study heterogeneity in Section 6.

Based on the assumed values of $\alpha_{i},\beta_{i},\gamma_{i},$ and $\lambda_{i}$, and the aggregate data extracted, we can apply Equation (8) followed by Equation (6) to obtain an estimate of $\mathrm{var}\left({\hat{\lambda }}_{i}\right)$.

#### 4.1.2 Continuous covariate

The approach to estimate $\mathrm{var}\left({\hat{\lambda }}_{i}\right)$ for a continuous covariate is similar to that described for a binary covariate, and the aggregate data required from each trial publication are shown in Table 2. There is the added complexity of having to specify the assumed distribution of the continuous covariate. For simplicity, this might be assumed to be a normal distribution (with a different mean and SD for each trial), especially as the mean and SD of key continuous covariates are usually reported in a trial publication (in the table of trial participant characteristics). However, depending on the covariate and information available, other distributions (eg, uniform) might be sensible to consider, and indeed a different type of distribution could be used in each trial if necessary.

**TABLE 2:** Aggregate data required from each trial to implement the proposed power calculation for the interaction between treatment effect and a continuous covariate

Total participants in the trial ($n_{i})$
Total participants in control group ($n_{Ci})$
Total participants in treatment group ($n_{Ti})$
Number of outcome events in the control group ($e_{Ci})$
Number of outcome events in the treatment group ($e_{Ti})$
Characteristics to define the continuous covariate's assumed distribution (eg, mean and SD in each group)

Similar to the binary covariate setting, assumptions are needed about the values of parameters $\alpha_{i},\beta_{i},\gamma_{i},$ and $\lambda_{i}$. Again, without loss of generalisability, centering $z_{\mathrm{ij}}$ by its mean allows $\alpha_{i}$ to be approximated by the overall log‐odds of the outcome event in the control group in trial i, and $\beta_{i}$ can be approximated by the overall treatment effect (log odds ratio) in trial i. As before, we suggest to let $\gamma_{i}$ = 0 (or consider a range of values), and choose $\lambda_{i}$ after discussion with clinicians on what constitutes a minimally relevant interaction effect size.

Based on the assumed values of $\alpha_{i},\beta_{i},\gamma_{i},$ and $\lambda_{i}$, and the aggregate data extracted, we can obtain an estimate of $\mathrm{var}\left({\hat{\lambda }}_{i}\right)$ for each trial by calculating Fisher's unit information matrix as described in Section 3.2. This is implemented in our Stata and R code. Essentially, we mimic what occurs in software behind the scenes, post‐estimation of a logistic regression model. That is, for each trial separately, our code:

- generates a large dataset (eg, 1 million participants) that matches the trial aggregate data provided in terms of the proportion of participants in the treatment and control groups, the proportion with an outcome event in each group, and the distribution of z in each group (eg, a normal distribution with a specified mean and SD);
- calculates $I_{i}=E_{\left(x,z\right)}\left(B\right)$ conditional on the values of $\alpha_{i},\beta_{i},\gamma_{i},$ and $\lambda_{i}$ specified for that trial (Equation 9);
- uses Equation (6) to calculate $\mathrm{var}\left({\hat{\lambda }}_{i}\right)=I_{i}^{-1}\left(4,4\right)/n_{i}$.

### 4.2 Step 2: Estimate the variance of the summary treatment‐covariate from the planned IPD meta‐analysis

Step 1 produces S estimates of $\mathrm{var}\left({\hat{\lambda }}_{i}\right)$, one for each trial. The variance of the summary interaction estimate from an IPD meta‐analysis of these trials can then be estimated, depending on whether step 1 assumed $\lambda_{i}$ was common or random across trials. In particular, when assuming $\lambda_{i}$ is common ($\lambda_{i}=\lambda )$, we can use Equation (5) to calculate the anticipated estimate of $\mathrm{var}\left(\hat{\lambda }\right)$ for the IPD meta‐analysis project:

$$\mathrm{var}\left(\hat{\lambda }\right)=\frac{1}{\sum_{i=1}^{S}{\left(\mathrm{var}\left({\hat{\lambda }}_{i}\right)\right)}^{-1}}.$$

### 4.3 Step 3: Calculate the power of the planned IPD meta‐analysis

The final step is to calculate the power of the planned IPD meta‐analysis project to detect λ. Assuming a common interaction for all trials, and based on a Wald‐test and a 5% statistical significance level, the power is approximately:

(10) $$\begin{array}{cc} & \text{Power}=\text{Prob}\left(\frac{\hat{\lambda }}{\sqrt{\mathrm{var}\left(\hat{\lambda }\right)}}>1.96\right)+\text{Prob}\left(\frac{\hat{\lambda }}{\sqrt{\mathrm{var}\left(\hat{\lambda }\right)}}<-1.96\right)\\ & \;=\Phi \left(-1.96+\frac{\hat{\lambda }}{\sqrt{\mathrm{var}\left(\hat{\lambda }\right)}}\right)+\Phi \left(-1.96-\frac{\hat{\lambda }}{\sqrt{\mathrm{var}\left(\hat{\lambda }\right)}}\right). \end{array}$$

Here, Φ(x) is the probability of sampling a value < x from a standard normal distribution, $\mathrm{var}\left(\hat{\lambda }\right)$ is the estimated variance of the summary interaction estimate (as obtained in step 2), and $\hat{\lambda }$ can be replaced with the assumed true λ (as defined in step 1). The power estimate is usually multiplied by 100 and reported as a %.

## 5 APPLIED EXAMPLES

We now apply our proposed method to two examples.

### 5.1 Example 1: Efficacy of beta‐adrenergic‐antagonist drugs in the prevention of gastrointestinal bleeding

Our first example considers the power of an IPD meta‐analysis conducted by Poynard et al,^18^ and is an example considered in previous methodology papers.^3^, ^8^ The project aimed to examine the efficacy of beta‐adrenergic‐antagonist drugs in the prevention of gastrointestinal bleeding for patients with cirrhosis and esophageal varices. IPD were obtained from four randomized trials involving a total of 286 patients randomized to active treatment and 383 to a control (placebo), and aggregate data from the trials are shown in Table 3.

**TABLE 3:** Aggregate data from 4 randomized trials included in the IPD meta‐analysis project of Poynard et al,^18^ as reported by Kovalchik and Cumberland^8^

Trial	Total participants (events) control	Total participants (events) treatment	Age in years: mean (SD) control	Age in years: mean (SD) treatment	Male, % control	Male, % treatment
1	112 (30)	118 (19)	54 (11)	54 (9)	71	71
2	89 (28)	85 (16)	53 (11)	55 (11)	73	67
3	49 (11)	30 (1)	55 (9)	53 (7)	71	73
4	53 (13)	53 (13)	57 (12)	55 (11)	76	74

We now ask the question: assuming this aggregate data could be obtained in advance (eg, from trial publications or investigators), what is the estimated power of a planned IPD meta‐analysis to examine treatment‐covariate interactions? We focus on the covariates sex and age, and use the three‐step process described in Section 4 to undertake our power calculations. As in previous considerations of this meta‐analysis,^8^ we assume λ = log(1.3) for the treatment‐sex interaction and λ = log(1.027) for the treatment‐age interaction. These correspond to an odds ratio that is 30% higher for males compared to females (ie, the treatment is less effective for males compared to females), and an odds ratio that is 30% higher for every 10‐year increase in age. We also assume that the interaction is common (the same) for all trials. Age is also assumed normally distributed in each trial, with a mean and SD as given in the table.

The results of the power calculation are shown in Table 4, assuming that the prognostic effect of the covariates is zero ($\gamma_{i}$ = 0). The power is low for both covariates. There is a power of 8.8% to detect the assumed treatment‐sex interaction, and a power of 26% to detect the assumed treatment‐age interaction. The power estimates barely change when allowing for a moderate prognostic effect of each covariate. For example, when assuming the prognostic effect of sex is an odds ratio of 1.5 ($\gamma_{i}$ = ln(1.5)), the power is 9.0%, compared to 8.8% when assuming there is no prognostic effect. Given the low power, it raises considerable doubts as to the value of doing this IPD meta‐analysis project in terms of reliably estimating the interactions. In particular, if known in advance of IPD collection, the low power might persuade researchers and funders that it is not worth their investment; or perhaps it may provide incentive to pursue IPD from additional trials, if they exist.

**TABLE 4:** Results of our power calculation for the Poynard example, using the three‐step process described in Section 3 based on the aggregate data shown in Table 3

	Variance estimate of each trial's interaction estimate ($\mathrm{var}\left({\hat{\lambda }}_{i}\right))$		Power (%) based on each trial separately		Weight (%) in the planned IPD meta‐analysis	
Study	Sex	Age	Sex	Age	Sex	Age
Trial 1	0.512	0.00115	6.55	12.06	41.17	35.05
Trial 2	0.596	0.00107	6.33	12.62	35.36	37.74
Trial 3	5.513	0.0229	5.14	5.34	3.82	1.76
Trial 4	1.073	0.00159	5.74	10.09	19.65	25.45

	Variance estimate of summary interaction estimate ($\mathrm{var}\left(\hat{\lambda }\right))$		Power (%) of planned IPD meta‐analysis	
Planned IPD meta‐analysis	Sex	Age	Sex	Age
All 4 trials	$\mathrm{var}\left(\hat{\lambda }\right)$ = 0.211	$\mathrm{var}\left(\hat{\lambda }\right)$ = 0.0004	**8.82**	**25.66**

*Note*: Assuming $\alpha_{i}$ is the observed log‐odds of the outcome event in the control group in trial i, $\beta_{i}$ is the observed overall treatment effect (log odds ratio) in trial i, there is no prognostic effect of the covariate ($\gamma_{i}=0)$ and λ is log(1.3) for sex (males compared to females) and log(1.3) for a 10‐year increase in age.

The anticipated contribution of each of the trials is similar for trials 1, 2, and 4, as revealed by the power estimates when taking each trial in isolation, and the anticipated percentage weight in the IPD meta‐analysis (Table 4). Trial 3 has the fewest number of participants and events, and the lowest SD of age, and so contributes relatively little; indeed, the percentage weight toward the summary interaction is just 3.82% and 1.76% for sex and age, respectively. It is clear that trial 3 basically provides no information towards the power calculation, which is unsurprising given the small sample sizes and sparse events, and so obtaining IPD from this trial is of low priority if the focus is solely on the treatment interaction with age or sex (but there may be other objectives that still warrant prioritizing the IPD for this trial).

We also compared our power estimates to those calculated using the approach of Kovalchik and Cumberland.^8^ Recall, their method uses a more approximate approach that (in addition to within‐trial information) also allows across‐trial information to contribute toward the power calculation, which is not recommended in practice due to potential for aggregation bias.^4^ However, in this example, the power estimates using Kovalchik and Cumberland's approach are 8.7% for sex and 26% for age, almost identical to our estimates of 8.8% and 26% based solely on within‐trial information. The similarity is because the across‐trial information is negligible in this example, as the mean covariate values vary only slightly across trials, as shown in Table 3. The percentage male varies only from 71% to 76%, and the mean age varies only from 53 to 57 years.

### 5.2 Example 2: Examining effect modifiers for exercise interventions to improve pain in osteoarthritis

In the Subgrouping and TargetEd Exercise pRogrammes for OsteoArthritis (STEER OA) project, IPD was obtained from existing randomized trials to examine potential participant‐level characteristics that interact with the effect of exercise interventions among people with knee and/or hip OA.^19^ Although pain and function outcomes were mostly analyzed on a continuous scale, one binary outcome of interest was whether the patient had a reduction of pain (compared to baseline pain) by 3‐6 months. Ultimately, 31 trials provided their IPD, and here we retrospectively consider the potential power of this IPD meta‐analysis for examining whether age or sex modify the effect of exercise for this binary outcome. The aggregate data for each trial are provided in Table 5, in terms of the total participants and outcome events for each treatment (exercise program) and control group, and the summary characteristics of age (mean, SD) and sex (percentage male).

**TABLE 5:** Aggregate data from 31 randomized trials included in the IPD meta‐analysis project of STEER‐OA,^19^ where events correspond to patients reporting to being in pain

Trial	Total participants (events) control	Total participants (events) treatment	Age in years: mean (SD) control	Age in years: mean (SD) treatment	Male, % control	Male, % treatment
1	68 (39)	142 (88)	64.25 (12.21)	65.29 (11.46)	22.06	30.99
2	24 (9)	24 (15)	67.17 (8.13)	65.17 (6.72)	20.83	37.50
3	44 (18)	45 (30)	64.59 (7.55)	64.52 (9.05)	54.55	48.89
4	99 (40)	100 (64)	62.54 (5.36)	60.96 (5.92)	30.30	40.00
5	74 (40)	148 (75)	62.28 (6.77)	63.93 (9.39)	36.49	28.38
6	159 (55)	153 (56)	69.63 (6.26)	69.86 (6.82)	37.74	36.60
7	63 (22)	63 (40)	64.94 (9.43)	63.21 (8.38)	26.98	22.22
8	41 (19)	111 (83)	69.61 (6.10)	70.39 (6.28)	17.07	29.73
9	43 (16)	45 (28)	60.37 (9.91)	61.76 (9.49)	46.51	24.44
10	16 (9)	23 (8)	75.19 (4.58)	73.57 (7.3)	25.00	21.74
11	108 (60)	109 (73)	68.23 (7.98)	67.94 (8.54)	35.19	34.86
12	23 (10)	25 (7)	61.30 (7.06)	65.03 (8.91)	26.09	12.00
13	35 (13)	36 (29)	61.53 (7.80)	63.34 (9.55)	31.43	33.33
14	49 (20)	56 (32)	65.20 (5.73)	65.43 (5.27)	12.24	21.43
15	140 (71)	278 (193)	66.76 (8.72)	66.52 (8.25)	31.43	28.78
16	147 (78)	71 (50)	59.11 (9.99)	57.79 (10.42)	59.18	59.15
17	54 (16)	53 (33)	63.56 (8.73)	65.62 (8.21)	46.30	43.40
18	78 (44)	80 (40)	68.61 (6.13)	69.02 (6.55)	NA	NA
19	40 (17)	40 (26)	58.20 (4.26)	57.25 (3.98)	NA	NA
20	44 (29)	43 (30)	63.91 (2.36)	63.81 (2.41)	0	0
21	9 (6)	19 (13)	70.44 (7.83)	66.37 (5.55)	44.44	47.37
22	11 (5)	21 (16)	71.18 (5.25)	72.43 (6.52)	9.09	14.29
23	54 (27)	55 (38)	57.21 (9.82)	58.4 (10.00)	48.15	43.64
24	54 (21)	55 (23)	68.60 (6.87)	66.85 (7.41)	27.78	34.55
25	20 (10)	20 (15)	67.10 (5.36)	66.14 (8.74)	35.00	5.00
26	17 (11)	17 (10)	70.76 (4.71)	69.59 (6.74)	23.53	23.53
27	102 (66)	101 (64)	66.59 (9.56)	64.18 (8.52)	45.10	37.62
28	156 (78)	235 (110)	61.89 (9.59)	61.54 (9.58)	35.90	36.17
29	27 (16)	28 (20)	78.93 (8.30)	78.89 (6.91)	33.33	21.43
30	102 (55)	98 (77)	67.78 (9.22)	68.29 (8.45)	20.59	22.45
31	23 (4)	23 (7)	66.78 (7.27)	67.57 (7.86)	52.17	60.87

For simplicity, we assume the same interaction sizes are of interest as in the previous example (λ = log(1.3) for the treatment‐sex interaction and λ = log(1.027) for the treatment‐age interaction), and that the true interaction is the same in each trial. We also assume the prognostic effect of age and sex is zero, and that age is normally distributed. Applying the three‐step process described in Section 4 to undertake our power calculations, we find the planned IPD meta‐analysis project has a power of 90.4% for age and 41.9% for sex (Table 6). Hence, the power is reassuringly high for age, but only moderate for sex. The power for sex is partly hampered by sex not being recorded for two trials (trials 18 and 19), and only females being recruited in another (trial 20), which means these three trials provide no information about the treatment‐sex interaction.

**TABLE 6:** Results of our power calculation for the STEER‐OA example, using the three‐step process described in Section 3 based on the aggregate data shown in Table 5

	Variance estimate of each trial's interaction estimate ($\mathrm{var}\left({\hat{\lambda }}_{i}\right))$		Power (%) based on each trial separately		Weight (%) in the planned IPD meta‐analysis	
Study	Sex	Age	Sex	Age	Sex	Age
Trial 1	0.50	0.0006	6.60	17.86	4.49	10.02
Trial 2	1.88	0.0067	5.42	6.19	1.19	0.97
Trial 3	0.80	0.0029	5.99	7.75	2.79	2.21
Trial 4	0.39	0.0027	7.05	7.96	5.74	2.38
Trial 5	0.37	0.0015	7.16	10.38	6.04	4.28
Trial 6	0.24	0.0013	8.41	11.12	9.49	4.86
Trial 7	0.78	0.0018	6.02	9.52	2.87	3.62
Trial 8	0.95	0.0039	5.84	7.07	2.36	1.67
Trial 9	0.94	0.0021	5.84	8.82	2.37	3.06
Trial 10	2.43	0.0158	5.33	5.50	0.92	0.41
Trial 11	0.36	0.0012	7.22	11.92	6.21	5.48
Trial 12	2.64	0.0061	5.30	6.31	0.85	1.06
Trial 13	1.46	0.0040	5.54	6.98	1.53	1.60
Trial 14	1.24	0.0052	5.64	6.52	1.81	1.23
Trial 15	0.22	0.0006	8.61	18.06	10.05	10.18
Trial 16	0.41	0.0009	6.96	13.97	5.48	7.06
Trial 17	0.70	0.0024	6.13	8.34	3.20	2.68
Trial 18	‐	0.0026	‐	8.11	‐	2.50
Trial 19	‐	0.0126	‐	5.63	‐	0.51
Trial 20	‐	0.0371	‐	5.21	‐	0.17
Trial 21	3.06	0.0161	5.26	5.49	0.73	0.40
Trial 22	6.88	0.0196	5.11	5.40	0.33	0.33
Trial 23	0.67	0.0017	6.19	9.87	3.35	3.89
Trial 24	0.71	0.0031	6.11	7.61	3.13	2.11
Trial 25	7.32	0.0106	5.11	5.75	0.31	0.61
Trial 26	2.85	0.0171	5.28	5.46	0.78	0.38
Trial 27	0.37	0.0011	7.18	12.62	6.12	6.02
Trial 28	0.19	0.0005	9.35	22.63	12.05	13.65
Trial 29	1.84	0.0059	5.43	6.34	1.22	1.09
Trial 30	0.64	0.0013	6.25	11.07	3.51	4.82
Trial 31	2.05	0.0091	5.39	5.87	1.09	0.71

Planned IPD meta‐analysis	Variance estimate of summary interaction estimate ($\mathrm{var}\left(\hat{\lambda }\right))$		Power (%) of planned IPD meta‐analysis	
	Sex	Age	Sex	Age
All trials	0.022	0.000064	**41.88**	**90.42**

*Note*: Assuming $\alpha_{i}$ is the observed log‐odds of the outcome event in the control group in trial i, $\beta_{i}$ is the observed overall treatment effect (log odds ratio) in trial i, there is no prognostic effect of the covariate ($\gamma_{i}=0)$ and λ is log(1.3) for sex (males compared to females) and log(1.3) for a 10‐year increase in age. Bold values are the key results.

The anticipated percentage weight for each trial is also revealing (Table 6) as it showcases which trials are most important for obtaining their IPD. For example, in terms of the age covariate, trials 1, 15, and 28 are each expected to provide over 10% of the weight toward the summary meta‐analysis result. Indeed, repeating our power calculation just using these three trials gives a power of 48% for age, which is over half the power of the full IPD meta‐analysis of all 31 trials. Hence, collecting the IPD from trials 1, 15, and 28 should be prioritized, in terms of examining age.

Also note that, as emphasized in Section 4, the power contribution of each trial depends on the covariate distribution and variance, not just the total number of participants and events. For example, although trial 4 has 199 participants and 104 events, and is thus one of the larger trials, it has a relatively low SD for age, and this leads to an anticipated percentage weight of only 2.4% toward the IPD meta‐analysis examining the treatment‐age interaction.

In contrast to the previous example, our power estimates are much lower than the approximate estimates obtained using the approach of Kovalchick and Cumberland,^8^ for both sex (41.9% vs 46.9%) and age (90.4% vs 95.3%). Recall that the approach of Kovalchick and Cumberland also allows across‐trial information to contribute toward the power calculation, which here is influential as the mean covariate value varies considerable across trials (unlike in the first example), as seen in Table 5. For example, the percentage male ranges from about 9% to 59%, and the mean age ranges from about 57 to 75 years.

## 6 EXTENSIONS

### 6.1 Allowing for heterogeneity

To allow for between‐trial heterogeneity in a treatment‐covariate interaction, we can extend the power calculation to,

(11) $$\text{Power}=T\left(-t_{S-1,0.975}+\frac{\hat{\lambda }}{\sqrt{\mathrm{var}\left(\hat{\lambda }\right)}}\right)+T\left(-t_{S-1,0.975}-\frac{\hat{\lambda }}{\sqrt{\mathrm{var}\left(\hat{\lambda }\right)}}\right),$$

where T(x) is the probability of sampling a value < x from a *t*‐distribution with a mean of zero and S−1 degrees of freedom, and S is the number of trials assumed to provide their IPD. Crucially $\mathrm{var}\left(\hat{\lambda }\right)$ must be derived from the random‐effects solution of Equation (5), and so an assumed value of $\hat{\tau }$ (the between‐trial SD of interactions) must also be given, which is not straightforward to ascertain. A *t*‐distribution is used (rather than a normal distribution) to help reflect extra uncertainty due to $\hat{\tau }$ being estimated in practice (akin to the use of the *t*‐distribution in the Hartung‐Knapp‐Sidik‐Jonkman [HKSJ] approach for deriving 95% confidence intervals after fitting a random‐effects meta‐analysis^20^, ^21^).

For example, returning to the STEER‐OA example from Section 5, and considering the power to detect a treatment‐age interaction of λ = log(1.027), let us apply Equation (11) (rather than Equation (10)) to allow for between‐study heterogeneity, which we assume is τ=0.015, as this would reflect quite large variability relative to the summary interaction of 1.027, such that the interaction may be negligible in some settings. The power is estimated to be 81.9%, which is somewhat lower than when assuming a common‐effect (90.4%), as expected, but still reassuringly high.

Equation (11) is only an approximation, and this is of most concern when there are a small number of trials and the true τ is close to zero, as then τ is particularly poorly estimated in practice (often with upward bias). A simulation‐based approach would be a better reflection of the uncertainty in that situation.^5^

### 6.2 Focusing on precision rather than power

In addition to (or even instead of) calculating power, researchers may also want to check the potential precision of their planned IPD meta‐analysis, in terms of whether confidence intervals will be sufficiently narrow to inform clinical decision‐making. For example, assuming no between‐trial heterogeneity in the treatment‐covariate interaction, an anticipated 95% confidence interval for the interaction from an IPD meta‐analysis can be calculated using $\hat{\lambda }\pm \left(1.96\times \sqrt{\mathrm{var}\left(\hat{\lambda }\right)\;}\right)$, where $\hat{\lambda }$ is the assumed interaction and $\mathrm{var}\left(\hat{\lambda }\right)$ is taken from Step 2 of our approach.

## 7 DISCUSSION

Since IPD meta‐analysis projects with large power are more likely to detect important effects of interest, they should (other things being equal) be a higher priority for funding. Therefore, careful assessments of power and sample size are an important part of planning and commissioning IPD meta‐analysis projects that aim to estimate a particular effect. In this article, we have proposed a new approach for calculating the power when designing an IPD meta‐analysis project aiming to pool randomized trials to estimate a treatment‐covariate interaction for a binary outcome. Our three‐step approach uses an asymptotic solution for calculating variances of interaction estimates, which is more exact compared to previous suggestions. Further, we showed how routinely reported aggregate data from trial publications can be used to inform the power calculation, to ensure the power is conditional on the actual characteristics (eg, sample size in each group, number of events, covariate distributions) of each trial for which IPD is promised or desired. This requires the distribution of the covariate to be assumed, with is potentially difficult for continuous covariates, for which we recommended using either a normal distribution or uniform distribution as a pragmatic approach.

We also showed how power calculations reveal which trials contribute most to the power, which could be used alongside other information (eg, relevance of trial population, outcome definitions, follow‐up length, and so forth), to inform decisions regarding which trials are prioritized for obtaining their IPD. This last point might be viewed as contentious, as some may argue that IPD should always be sought from all trials. The counterargument is that IPD meta‐analysis projects are time‐consuming (and thus costly), and sometimes limited resources are available or there is an urgent need for quick answers (eg, in a pandemic); then, it may be justifiable to focus efforts on obtaining IPD from the subset of trials that provide the most information to answer the research question reliably and in the shortest time‐frame for the population of interest.

As with any sample size calculation, our approach is a pragmatic one to help gauge potential power under plausible assumptions. In practice, the actual power (and confidence interval width of results) of the IPD meta‐analysis project will change if the modeling assumptions are incorrect (eg, in terms of the amount of IPD actually received from existing trials, the prognostic effect of the covariate, the assumed distribution of the continuous covariate, etc.). Thus, funding applications for IPD meta‐analysis projects might display a range of power calculations (or expected precision of estimates) conditional on a range of assumptions. For example, they might estimate power (or expected precision) when optimistically assuming IPD will be obtained from all trials, but also when cautiously assuming IPD will only be provided by trials that already provided verbal or written agreement.

In summary, we have provided a new approach for estimating the potential power or precision of an IPD meta‐analysis project aiming to estimate a treatment‐covariate interaction with a binary outcome from randomized trials. We hope the approach, and our associated Stata and R code, improves the uptake of power calculations in future IPD meta‐analysis projects, to help researchers and funders gauge the likelihood of success. In the near future we will also launch a dedicated package called *ipdmapower*, to implement this and other power calculations for IPD meta‐analysis projects.

## FUNDING INFORMATION

Richard D. Riley, Miriam Hattle, Rebecca Whittle, Gary S. Collins, and Joie Ensor were supported by funding from the MRC Better Methods Better Research panel (Grant reference: MR/V038168/1). Gary S. Collins was supported by the NIHR Biomedical Research Centre, Oxford, and Cancer Research UK (program Grant: C49297/A27294).

## Supporting information


**Appendix S1** Supporting information

## Data Availability

The work presented involves applying equations using aggregated or simulated data, and therefore no actual individual‐level data is available for sharing. Simulation and example code is provided in the Supplementary Material, and aggregate data for examples are shown in the paper itself.
