# Supplementary material for: Calculating the power to examine treatment‐covariate interactions when planning an individual participant data meta‐analysis of randomized trials with a binary outcome
Source: Stat Med. 2022 Aug 5;41(24):4822–37. doi: 10.1002/sim.9538 (PMC9805219; doi:10.1002/sim.9538)
Supplement: Supplementary file 1 — Appendix S1 Supporting information [file SIM-41-4822-s001.zip › Supplementary Material.docx]

## Supplementary Material

***Stata code***

Stata code and aggregate data for the Poynard example are provided as additional files, for each of the age and sex applications. Also provided are the Stata codes for the simulation studies evaluating the performance of the proposed approach to estimate the variance of an interaction estimate for a binary or continuous covariate in a single trial.

***Distribution of age in the trial of Wilson et al***

1. Usual care group

1. Intervention group
